# Typhoid Pneumonia

**Published:** 1893-02

**Authors:** B. Le Baron Baylies

**Affiliations:** Brooklyn, N. Y.


### TYPHOID PNEUMONIA.

B. LE BARON BAYLIES, M. D., BROOKLYN, N. Y.

November 5th, 1890.—James Brown, printer; dark complexioned; about six feet in height; thirty years of age. Arrived three days ago from California, a cough attacking him on the train. Since his arrival, though fatigued and feeble, he had traveled much about town, and last evening, at 6 P. M., had a chill, followed by hot fever, which still continued November 5th, 5.30 P. M. Face dark-hued, cheeks flushed; constantly sleeping till awakened by cough, which is attended by pain in the right upper chest and shoulder. When addressed aloud, he answers clearly, and falls asleep again immediately. He had a stupid feeling in the head, and sleepiness during the entire chill. Now, his *head feels big;* his hands and fingers *big and heavy*—"a ton weight," he says, as if impossible to be moved, but "they get limbered up" after a little effort. He wants to turn, as he becomes uneasy from lying long in one position; the shoulders and back feel sore when long lain upon. At present, he lies partially on the right side, face inclined toward the pillow, supporting himself upon the left elbow, knees slightly bent. Temperature of the body high; rapid pulse and respiration. Physical signs indicate pneumonia of the upper portion of the right lung; pneumonic sputa, of the first stage.

The sore feeling of the parts of the body in contact with the bed, especially directs attention to Arnica and Baptisia; the stupor and the sensation of enlargement and weight of the head, hands, and fingers elect Baptisia as the remedy, of which the 1000th was given in solution every two hours; milk, taken freely, about every two hours. The patient is a vegetarian, and refuses beef broth or beef tea.

November 5th, 8 P. M.—Very restless; temperature, 105.6°; pulse, 120; respiration, 35. The whole body aches.

November 6th, 2 A. M.—Temperature, 104.2°; pulse, 120; respiration, 35. Perspiring a little on the forehead.

November 6th, 6 A. M.—Temperature, pulse, and respiration the same as four hours earlier. Very little sleep during the night.

November 6th, 9 A. M.—Temperature, 104°; pulse and respiration unchanged.

November 6th, 5 P. M.—Temperature, 105.4°; pulse reduced six beats, to 114; respiration, raised 5, to 40.

November 6th, 6.30 P. M.—Temperature, 102.2°, after an alcohol bath—*i. e.*, after sponging the body with alcohol and water—has taken hitherto the milk warm; now cold—a cupful at a time.

Baptisia $^{1000}$ in solution every three hours.

November 7th, 5 A. M.—Temperature, 102°; pulse, 114; respiration, 40.

November 7th, 8 A. M.—Temperature, 103.6°; pulse, 114; respiration, 38. He has slept some and is less restless. Sputa, orange colored; uniform in density.

November 7th, 2 P. M.—Had less aching and soreness to-day. Temperature, 104°; pulse, 116; respiration, 38. This rise of temperature was due to the obtrusion of a talkative friend.

November 7th, 5 P. M.—Perspiring very freely, and in evening very comfortable. At midnight, restful.

November 8th.—Took for the first time a little oatmeal-gruel in the morning.

November 8th, 6 P. M.—Very comfortable; without pain; sleeping; has had slight nose-bleed; is very irritable; complains of weakness, of exhaustion.

November 8th, 9 P. M.—Temperature, 103.6°; pulse, 100; respiration 32; has been very comfortable and quiet, except when waking with cough, which he says weakens him; mind quite clear and bright; enjoys his food.

November 9th, 7 P. M.—Temperature, 104°; pulse, 100; respiration, 34; he complains of sharp stitching in the right hypochondrium when coughing; the pain lasting half an hour.

November 9th, 9 P. M.—Quietly sleeping; general perspiration.

November 10th, 2 A. M.—Temperature, 103.2°; pulse, 104; respiration, 36. Feeling comfortable.

November 10th, 7 A. M.—Has slept five to six hours during the past night. Temperature, 103.4°; pulse, 98; respiration, 45.

November 10th, 10 P. M.—Reduced the dose of Baptisia$^{1000}$ by diluting the solution with water twenty times, giving it only during the febrile aggravation.

November 11th, 1.30 A. M.—Very comfortable.

November 11th, 8 A. M.—Temperature, 100.4°; pulse, 76; respiration, 30. Slept comfortably, at intervals, five to six hours during the night; perspiring freely.

November 11th, 11 A. M.—Temperature, 98.6°; respiration, 35. Medicine omitted.

November 11th, 2 P. M.—Temperature, 98.5°; pulse, 70; respiration, 30.

November 11th, 6.15 P. M.—Temperature, 99.2°; pulse, 70; respiration, 30. Has been very comfortable all day, little cough, causing less shock; sputa nearly white, skin of the arms and hands is natural, no inclination or ability to pass urine, which has been drawn three times since the morning of the 10th (night and morning). He continues to take about half a pint of cold milk every two hours.

November 12th, 3 A. M.—Temperature, 98.1°; pulse, 66; respiration, 28. No medicine.

November 12th, 5.30 P. M.—Temperature, 98.4°; pulse, 66; respiration, 28.

November 13th, 2 A. M.—A large, solid fœcal movement; quiet sleep for three and a half hours; passed urine naturally, with one exception, since the 11th.

November 19th.—Temperature, pulse, and respiration normal.

November 21st.—Well, except occasional slight cough and some debility, from all of which he soon recovered with no further treatment, except one dose of Sulphur$^{200}$ and one of Kali-carb.$^{45m}$ while convalescent.

On the second day of this man's illness, the nurse being relieved for a short time, visited the old-school hospital where she was being trained, and seeing the physician in charge, was asked: "Well, what ails your patient?"

A. "Typhoid pneumonia (naming the temperature, state of the pulse, and respiration)."

Q. "What is the doctor doing? Giving Quinine—Antipyrine—using the cold bath?"

A. "No, only giving a little of something in water every two hours."

Doctor. "Well, *he* won't *live!*"

A few days later she saw the same physician, and reported the patient's improvement.

"Well, it wasn't pneumonia!"

"Yes, but it was!" said the nurse; "with painful cough and raising bloody phlegm."

"A very remarkable case," was the reply.

